# One-pot synthesis and in-vitro anticancer evaluation of 5-(2′-indolyl)thiazoles

**DOI:** 10.1038/srep23401

**Published:** 2016-03-29

**Authors:** Buchi Reddy Vaddula, Mukund P. Tantak, Rachana Sadana, Michael A. Gonzalez, Dalip Kumar

**Affiliations:** 1 Sustainable Technology Division, National Risk Management Research Laboratory, U.S. Environmental Protection Agency, 26 West Martin Luther King Drive, MS 483, Cincinnati, OH 45268, USA; 2 Department of Chemistry, Birla Institute of Technology and Science, Pilani 333 031, India; 3 Department of Natural Sciences, University of Houston – Downtown, Houston, TX 77002, USA

## Abstract

A series of 5-(2′-indolyl)thiazoles were synthesized and evaluated for their cytotoxicity against selected human cancer cell lines. The reaction of thioamides **3** with 3-tosyloxypentane-2,4-dione **4** led to *in situ* formation of 5-acetylthiazole **5** which upon treatment with arylhydrazines **6** in polyphosphoric acid resulted in the formation of 5-(2′-indolyl)thiazoles **2**. Among the synthesized 5-(2′-indolyl)thiazoles, compounds **2d–f**, and **2h** exhibited encouraging anticancer activity and also selectivity towards particular cell lines (IC_50_ = 10–30 μM). Further studies on the SAR of compound **2e** may result in good anticancer activity.

Multicomponent reactions (MCRs) are one-pot multi-step reactions comprising three or more substrates. They are popular in drug discovery for being important tools in rapidly generating a library of novel compounds with diverse substitutions and molecular complexity^1^. The advantages associated with MCRs include atom economy, simple operation, and high yields of the products along with eliminating the need for isolation of intermediates. Additionally, the application of microwaves in organic synthesis has enabled the chemists to accelerate reactions, improve yields, and easily achieve desired products^2^,^3^,^4^.

Indole alkaloids extracted from plants and marine sources are well known for their chemistry and biology. The biological significance of these naturally available alkaloids secured much attention resulting in exploring the structural novelty of these molecules. 5-(2^′^-Indolyl)azoles **2** possess an indole ring at the C-5 position of azole ring as in 5-(3^′^-indolyl)azoles **1,** but is connected at the second position of indole unlike the third position in natural derivatives (Fig. 1).

Thiazole derivatives are one of the most revered compounds with many applications and biological activities attributed to them. Indolylthiazole and its related heterocyclic systems are found in many natural products. The anticancer compounds, Camalexin^5^,^6^ and the naturally occurring BE 10988, are thiazole-substituted indole derivatives^7^. Additionally, the thiazole and benzothiazole derivatives of indole have been found to exhibit broad therapeutic activities including antimicrobial and antitumor^8^,^9^,^10^,^11^,^12^,^13^,^14^,^15^.

The naturally occurring bis(indolyl) alkaloids and their analogues, in general, have also exhibited significant anticancer activities^16^. For example, Nortopsentins and Topsentins represent a class of deep-sea sponge metabolites which display potent biological activities such as antitumor^17^, antiviral^18^, and antiinflammation^19^. Nortopsentin analogues, such as 2,4-bis(indolyl)thiazoles, also have exhibited good cytotoxicity against a panel of human tumor cell lines with GI_50_ values as low as 0.888 μM^13^. The closely related thiazolylbenzofuran derivatives^20^ have been shown to possess leukotriene and SRS-A antagonist or inhibitor activities. Our research group has prepared a series of indolylazoles such as 5-(3-indolyl)-1,3,4-oxadiazoles^21^, 4-(3-indolyl)oxazoles^22^, 5-(3-indolyl)-1,3,4-thiadiazoles^23^, and indolyl-1,2,4-triazoles^24^ as potential anticancer agents against human cancer cell lines.

Instigated by the role of indolylthiazoles and their bioisosteres in a variety of therapeutic activities as well as paucity of their one-pot preparation, we have contrived the synthesis of 5-(2′-indolyl)-2-substituted thiazoles **2** (Fig. 2). This one-pot synthesis was carried out by *in situ* generation of 5-acetylthiazole **5** from the reaction of thioamide **3** with 3-tosyloxypentane-2,4-dione **4** in ethanol with subsequent treatment of **5** with arylhydrazines **6** in polyphosphoric acid (PPA).

## Results and Discussion

**Figure 3:**
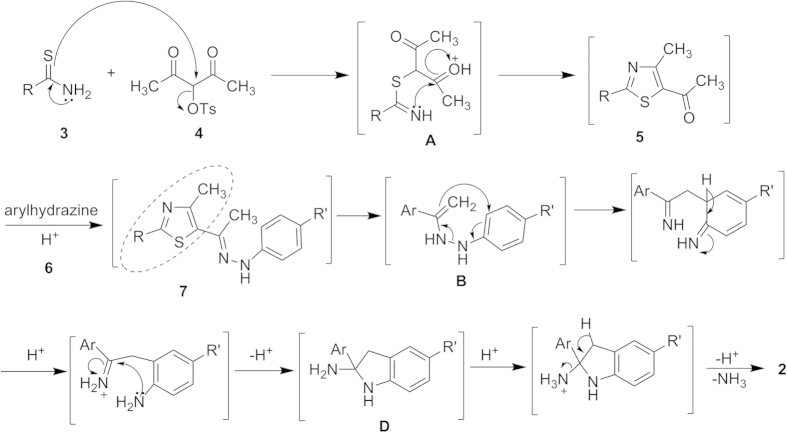
Plausible mechanistic pathway for the formation of indolylthiazoles 2.

The preparation of indolylthiazoles **2** was initiated with a trial reaction of 4-methoxybenzothioamide **3a** with 3-tosyloxypentane-2,4-dione **4** in ethanol. The reaction mixture was then refluxed at 80 °C for 4 hours in ethanol without any catalyst to afford 5-acetylthiazole **5a** in 90% yield. Upon confirming the synthesis of the 5-acetylthiazole **5a** by comparing its melting point with that of literature report^25^, it was further reacted with an equimolar quantity of phenylhydrazine (**6a**) in ethanol at 80 °C, to produce the corresponding hydrazone **7a** (*cf.* Fig. 3). The solid hydrazone **7a** obtained was then subjected to a Fischer indole cyclization under varying acidic conditions (Table 1). In most of the cases attempted, either the reaction failed to initiate or led to a complex mixture with trace amounts of the desired product. The cyclization was then attempted by refluxing the hydrazone **7a** in ethanol in the presence of HCl or *p*-toluenesulfonic acid (*p*-TsOH) or phosphotungstic acid (PTA). No desired product was formed under these reaction conditions even after extended heating (12 h).

The cyclization was also attempted by directly heating the hydrazone **7a** with phosphoric acid (H_3_PO_4_) or zinc chloride (ZnCl_2_) or conc. sulfuric acid (H_2_SO_4_) or formic acid (HCOOH) or PTA. Finally, the hydrazone **7a** was heated in PPA at 80 °C for 15 min to obtain the desired indolylthiazole **2a** in 30% yield. The structure of **2a** was confirmed by ^1^H NMR and mass spectral data. ^1^H NMR spectrum of **2a** showed characteristic singlets at δ 6.68 and δ 2.67 due to indole C3-H and C4 methyl of thiazole, respectively. The mass spectrum of **2a** showed the expected molecular ion peak [M + H]^+^ at *m/z* 321.1 which was found to be in agreement with the calculated value.

The reaction was further simplified by the direct reaction of phenylhydrazine **6a** with 5-acetyl-thiazole **5a** in presence of PPA. Encouraged by the outcome, a one-pot trial was attempted by subjecting equimolar quantities of 4-methoxybenzothioamide **3a** with 3-tosyloxypentane-2,4-dione **4** under microwave (MW) irradiation in ethanol at 80 °C for 10 min. Upon completion of the reaction, phenylhydrazine **6a** was added to the reaction vessel and exposed to MW irradiation at the same temperature for an additional 10 min. After the addition of PPA (two drops) the contents were further exposed to MW for 15 min to afford the desired indolylthiazole **2a** in 65% yield.

The developed protocol was extended to prepare various 5-(2′-indolyl)thiazoles **2b–j** using thioamides **3** and arylhydrazines **6** in good yields (65–85%). The 4-methoxythiobenzamide, 3,4,5-trimethoxythiobenzamide and 4-chlorothiobenzamide exhibited almost similar reactivity towards 3-tosyloxypentane-2,4-dione. However, thioacetamide and indolylthioamide were more reactive and afforded the corresponding products in relatively higher yields.

The thioacetamide and phenylhydrazine reacted to afford **2d** in 65% yield, whereas reaction involving indole-3-thioamide and phenylhydrazine afforded **2e** in 80% yield. The yields of the compounds **2f–j** were in the range of 70–80% demonstrating that substitutions on arylhydrazine have little effect on the formation of final products **2**. The reaction of 4-methoxythiobenzamide with phenylhydrazine and 4-chlorophenylhydrazine yielded **2a** and **2b** in 65% and 68% yields, respectively. Arylhydrazines **6** bearing an electronegative atom (Cl, Br and F) were equally reactive with 5-acetylthiazole **5,** whereas arylhydrazines **6** bearing an electron-donating group (4-OMe) were relatively more reactive to undergo the Fischer indole cyclization.

Formation of 5-(2′-indolyl)thiazoles **2** involves an initial nucleophilic displacement of a tosyloxy group in **4** by thioamide **3** to generate intermediate species **A** which undergoes cyclization with concomitant loss of water molecule to afford acetylthiazole **5**. Subsequent reaction of **5** with arylhydrazines **6** leads to hydrazones **7** which undergoes a [3,^3^]-sigmatropic rearrangement as depicted in Fig. 3 to produce 5-(2′-indolyl)thiazoles **2**.

Synthesized 5-(2′-indolyl)thiazoles compounds **2a–j** were then screened for their *in vitro* cytotoxicity against five human cancer cell lines: breast (BT-474, MCF-7 and MDA-MB-231, MDA-MB-157) and colon (HTC-116). Anticancer activity of 5-(2′-indolyl)thiazoles **2a–j** against cancer cell lines are expressed in terms of IC_50_ values (Table 2). Some of the compounds have shown moderate activity. Structure–activity relationship (SAR) study revealed that substitution at C-2 position of the thiazole ring is crucial for inducing cytotoxicity and selectivity against particular cancer cell line.

The presence of a 4-methoxyphenyl group at C-2 position and absence of any substituent on the C-5 indole ring led to compound **2a** not having any significant activity against the tested cancer cell lines (IC_50_ > 100 μM). Upon placing a chloro substituent on the indole ring of **2a** led to compound **2b** also without any improvement in activity (IC_50_ > 100 μM). The anticancer activity was further studied by replacing the 4-methoxyphenyl in **2a** with 4-chlorophenyl resulting in compound **2c** without any improvement in activity (IC_50_ > 100 μM). The presence of a methyl substituent at the C-2 position of thiazole **2d** exhibited selectivity towards breast cancer cell line, BT-474, with an improved activity (IC_50_ = 30 μM), but relatively moderate towards colon cancer cell line, HTC-116 (IC_50_ > 30 μM). The presence of a heterocyclic indole ring at the C-2 position resulted in bis(indolyl)thiazole **2e**, which closely resembles the naturally occurring bisindolyl alkaloids such as Nortopsentins. Compound **2e** exhibited very good selectivity and potency (IC_50_ = 10 μM) when compared to other compounds of the series.

It was earlier shown that one of the important structural units is a trimethoxyphenyl moiety in several known natural antimitotic agents (Combretastatin A-4, Colchicine, Podophyllotoxin and Steganacin) that binds at the Colchicine site of tubulin^26^,^27^. Incorporation of such a crucial structural feature of lead anticancer compounds into 5-(2^′^-indolyl)thiazoles **2** may results in a potent anticancer compound. Compounds **2f–j** were synthesized with a 3,4,5-trimethoxy phenyl substitution at C-2 position of the thiazole ring anticipating improved activity and selectivity towards a particular cell line. The compound **2f** with no substituents on the indole ring at C-5 of thiazole exhibited very good selectivity towards BT-474 breast cancer cell line (IC_50_ = 20 μM) whereas, the presence of a fluoro substituent on the indole ring (compound **2h**) could inhibit both BT-474 and MDA-MB-157 (IC_50_ = 30 μM). The other compounds exhibited moderate to insignificant activity. Further improvement in activity may be achieved to a great extent by investigating the compound **2e** with further substitutions on both indole rings.

## Conclusions

The synthesis of prominent 5-(2′-indolyl)thiazoles was achieved in one-pot using a sequential reaction of thioamides, 3-tosyloxypentane-2,4-dione and arylhydrazines. All the 5-(2′-indolyl)thiazoles were obtained in good yields. Overall, the protocol is appreciable in terms of the high exploratory power, short duration and ease of availability of the starting materials. The generality of this protocol is demonstrated by preparing a diverse library of 5-(2′-indolyl)thiazoles from various thioamides and arylhydrazines. The similitude of the 5-(2′-indolyl)thiazoles to naturally occurring indole-based alkaloids envisage their biological importance. The anticancer activity studied against breast and colon cancer cell lines show that the compounds **2d–f**, and **2h** exhibit encouraging anticancer activity and also selectivity towards particular cell lines (IC_50_ = 10–30 μM). Further studies on the SAR of **2e** may result in compounds with greatly improved anticancer activity.

## Experimental

### Representative procedure for the synthesis of 5-(2′-indolyl)thiazole (2a)

A mixture of 4-methoxybenzothioamide (2.0 mmol) and 3-tosyloxypentane-2,4-dione (2.0 mmol) in ethanol was subjected to MW irradiation (100 watt power) for 10 min at 80 °C temperature. Upon consumption of the thioamide as indicated by TLC, an appropriate phenylhydrazine (2.1 mmol) was added to reaction mixture and exposed to MW for 10 min at same temperature. Polyphosphoric acid (two-drops) was added to the reaction mixture and irradiated in MW for 15 min at 80 °C. The reaction contents were diluted with water and extracted with ethyl acetate (2 × 10 mL). The combined organic phase was dried over anhydrous sodium sulphate, concentrated in vacuo, and purified by passing through a column of silica-gel using ethyl acetate-hexane as eluent to afford the pure 5-(2′-indolyl)thiazole **2a** in 65% yield.

### Analytical data of the synthesised 5-(2′-indolyl)thiazoles 2

*5-(1H-Indol-2-yl)-2-(4-methoxyphenyl)-4-methylthiazole (**2a**).* Yield 45%. R_f_ = 0.51 (hexane/EtOAc, 6:4). m.p. 182–185 °C. ^1^H NMR (400 MHz, CDCl_3_) δ: 8.28 (s, br, 1H), 7.91–7.78 (m, 2H), 7.63 (d, *J* = 7.80 Hz, 1H), 7.41 (d, *J* = 8.0 Hz, 1H), 7.23 (dt, *J* = 7.08 and 1.12 Hz, 1H), 7.15 (dt, *J* = 7.88 and 0.92 Hz, 1H), 6.97–6.94 (m, 2H), 6.69–6.68 (m, 1H), 3.86 (s, 3H), 2.67 (s, 3H). MS (ESI): *m*/*z* [M + H]^+^ calcd for C_19_H_16_N_2_OS: 321.1; found: 321.1.

*5-(5-Chloro-1H-indol-2-yl)-2-(4-methoxyphenyl)-4-methylthiazole (**2b**).* Yield 48%. R_f_ = 0.59 (hexane/EtOAc, 6:4). m.p. 197–198 °C. ^1^H NMR (400 MHz, DMSO-*d*_*6*_) δ: 11.05 (s, 1H), 7.86 (d, *J* = 8.76 Hz, 2H), 7.51 (s, 1H), 7.37 (d, *J* = 8.52 Hz, 1H), 7.07 (dd, *J* = 8.48 and 1.84 Hz, 1H), 6.97 (d, *J* = 8.80 Hz, 2H), 6.56 (s, 1H), 3.86 (s, 3H), 2.65 (s, 3H). MS (ESI): *m*/*z* [M + H]^+^ calcd for C_19_H_15_ClN_2_OS: 355.1; found: 355.1.

*2-(4-Chlorophenyl)-5-(1H-indol-2-yl)-4-methylthiazole (**2c**).* Yield 55%. R_f_ = 0.61 (hexane/EtOAc, 6:4). m.p. 172–175 °C. ^1^H NMR (400 MHz, CDCl_3_) δ: 8.51 (s, br, 1H), 7.98–7.92 (m, 2H), 7.64 (d, *J* = 7.84 Hz, 1H), 7.46–7.42 (m, 3H), 7.17 (dt, *J* = 7.08 and 1.12 Hz, 1H), 7.15 (dt, *J* = 8.04 and 1.04 Hz, 1H), 6.73 (s, 1H), 2.74 (s, 3H). MS (ESI): *m*/*z* [M + H]^+^ calcd for C_18_H_13_ClN_2_S: 325.1; found: 325.2.

*5-(1H-Indol-2-yl)-2,4-dimethylthiazole (**2d**).* Yield 65%. R_f_ = 0.56 (hexane/EtOAc, 6:4). m.p. 238 °C. ^1^H NMR (400 MHz, DMSO-*d*_*6*_) δ: 11.38 (s, 1H), 7.56 (d, *J* = 7.9 Hz, 1H), 7.42 (dd, *J* = 8.1, 0.8 Hz, 1H), 7.17–7.11 (m, 1H), 7.04 (ddd, *J* = 7.9, 7.1 and 1.0 Hz, 1H), 6.65–6.62 (m, 1H), 2.70 (s, 3H), 2.53 (s, 3H). MS (ESI): *m*/*z* [M + H]^+^ calcd for C_13_H_12_N_2_S: 229.1; found: 229.2.

*5-(1H-Indol-2-yl)-2-(1H-indol-3-yl)-4-methylthiazole (**2e**).* Yield 60%. R_f_ = 0.32 (hexane/EtOAc, 6:4). m.p. 146–149 °C. ^1^H NMR (400 MHz, CDCl_3_) δ: 8.73 (s, 1H), 8.33 (s, 1H), 8.01 (s, 1H), 7.92 (s, 1H), 7.55 (s, 1H), 7.45–7.38 (m, 4H), 7.24–7.21 (m, 3H), 2.17 (s, 3H). MS (ESI): *m*/*z* [M + H]^+^ calcd for C_20_H_15_N_3_S: 330.1; found: 330.2.

*5-(1H-Indol-2-yl)-4-methyl-2-(3,4,5-trimethoxyphenyl)thiazole (**2f**).* Yield 55%. R_f_ = 0.30 (hexane/EtOAc, 6:4). m.p. 126–129 °C. ^1^H NMR (400 MHz, DMSO-*d*_*6*_) δ: 11.25 (s, 1H), 7.65 (d, *J* = 8.02 Hz, 1H), 7.38 (dd, *J* = 8.1 and 0.8 Hz, 1H), 7.17–7.13 (m, 1H), 7.05–7.03 (m, 1H), 6.95 (s, 2H), 6.64 (s, 1H), 4.02 (s, 6H), 3.93 (s, 3H), 2.80 (s, 3H). MS (ESI): *m*/*z* [M + H]^+^ calcd for C_21_H_20_N_2_O_3_S: 381.1; found: 381.1.

*5-(5-Chloro-1H-indol-2-yl)-4-methyl-2-(3,4,5-trimethoxyphenyl)thiazole (**2g**).* Yield 52%. R_f_ = 0.32 (hexane/EtOAc, 6:4). m.p. 126–127 °C. ^1^H NMR (400 MHz, DMSO-*d*_*6*_) δ: 11.25 (s, 1H), 7.51 (s, 1H), 7.37 (d, *J* = 8.52 Hz, 1H), 7.12 (s, 2H), 7.07 (dd, *J* = 8.48 and 1.84 Hz, 1H), 6.56 (s, 1H), 4.00 (s, 6H), 3.95 (s, 3H), 2.84 (s, 3H). MS (ESI): *m*/*z* [M + H]^+^ calcd for C_21_H_19_ClN_2_O_3_S: 415.1; found: 415.1.

*5-(5-Fluoro-1H-indol-2-yl)-4-methyl-2-(3,4,5-trimethoxyphenyl)thiazole (**2h**).* Yield 55%. R_f_ = 0.40 (hexane/EtOAc, 6:4). m.p. 128–129 °C. ^1^H NMR (400 MHz, DMSO-*d*_*6*_) δ: 11.38 (s, 1H), 7.62–7.61 (m, 1H), 7.48–7.51 (m, 1H), 6.90 (s, 2H), 7.11–7.09 (m, 1H), 6.59 (s, 1H), 4.02 (s, 6H), 3.97 (s, 3H), 2.87 (s, 3H). MS (ESI): *m*/*z* [M + H]^+^ calcd for C_21_H_19_FN_2_O_3_S: 399.1; found: 399.1.

*5-(5-Bromo-1H-indol-2-yl)-4-methyl-2-(3,4,5-trimethoxyphenyl)thiazole (**2i**).* Yield 58%. R_f_ = 0.41 (hexane/EtOAc, 6:4). m.p. 128–131 °C. ^1^H NMR (400 MHz, DMSO-*d*_*6*_) δ: 11.39 (s, 1H), 7.98–7.97 (m, 1H), 7.66–7.64 (m, 1H), 7.05 (s, 2H), 7.43–7.41 (m, 1H), 6.76 (s, 1H), 3.97 (s, 6H), 3.93 (s, 3H), 2.82 (s, 3H). MS (ESI): *m*/*z* [M + H]^+^ calcd for C_21_H_19_BrN_2_O_3_S: 458.0; found: 458.1 [M + H]^+^ and 460.1 [M + H + 2]^+^.

*5-(5-Methoxy-1H-indol-2-yl)-4-methyl-2-(3,4,5-trimethoxyphenyl)thiazole (**2j**).* Yield 50%. R_f_ = 0.43 (hexane/EtOAc, 6:4). m.p. 129–132 °C. ^1^H NMR (400 MHz, DMSO-*d*_*6*_) δ: 11.34 (s, 1H), 7.57–7.56 (m, 1H), 7.38–7.36 (m, 1H), 7.01 (s, 2H), 6.90–6.94 (m, 1H), 6.72 (s, 1H), 3.96 (s, 6H), 3.92 (s, 3H), 3.79 (s, 3H), 2.68 (s, 3H). MS (ESI): *m*/*z* [M + H]^+^ calcd for C_22_H_22_N_2_O_4_S: 411.1; found: 411.2.

### *In vitro* anticancer screening

Five human cancer cell lines (BT-474, MCF-7, MDA-MB-231, HTC-116, and MDA-MB-157) were cultured in RPMI 1640 media supplemented with 10% heat inactivated fetal bovine serum and 1% penicillin/streptomycin. They were seeded in 96-well plates at a density of 4 × 10^3^ cells per well for 12 h. Cells were incubated with various concentrations of the compounds ranging from 10 nM to 1 mM. After 48 h, MTT (3-(4,5-dimethylthiazol-2-yl)-2,5-diphenyltetrazolium bromide) was added to the final concentration of 0.2 mg/mL and incubated for 30 min. The cells were washed twice with PBS and lysed in 100 μL dimethylsulfoxide, and the absorbance was measured at 570 nm using Tecan Spectrafluor Plus.

## Additional Information

**How to cite this article**: Vaddula, B. R. *et al*. One-pot synthesis and in-vitro anticancer evaluation of 5-(2′-indolyl)thiazoles. *Sci. Rep.* **6**, 23401; doi: 10.1038/srep23401 (2016).

## Figures and Tables

**Figure 1:**
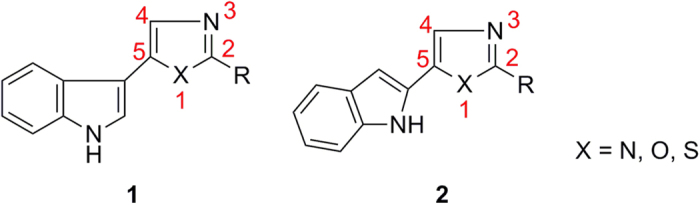
5-(3^′^-Indolyl)azoles 1 and 5-(2^′^-Indolyl)azoles 2.

**Figure 2:**
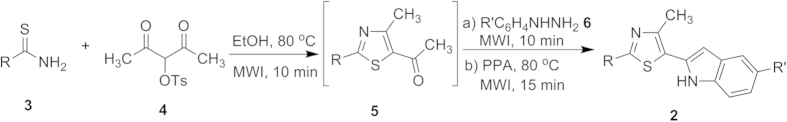
One-pot synthesis of 5-(2′-indolyl)thiazoles 2.

**Table 1:** Cyclization of hydrazone 7a.

S.No.	Reagent	Condition	Solvent	Yield (%)
1	HCl	Reflux	Ethanol	–
2	H_3_PO_4_ (6 M)	Heating (80 °C)	–	–
3	H_3_PO_4_ (18 M)	Heating (80 °C)	–	–
4	*p*-TsOH	Microwave	Neat	–
5	*p*-TsOH	Reflux	Ethanol	–
6	*p*-TsOH	Reflux	Acetonitrile	–
7	*p*-TsOH	Grinding	Neat	–
8	Conc. H_2_SO_4_	Heating (80 °C)	–	–
9	PPA	Room temp.	–	–
10	PPA	Heating (80 °C)	–	30
11	PPA	Reflux (110 °C)	Toluene	–
12	PPA	Microwave (80 °C)	Ethanol	65
13	ZnCl_2_	Grinding	Neat	–
14	ZnCl_2_	Microwave	Neat	–
15	ZnCl_2_	Reflux	Ethanol	–
16	AcOH	Heating (116 °C)	–	–
17	PTA	Reflux (80 °C)	Ethanol	–
18	PTA	Grinding	–	–
19	HCOOH	Heating (80 °C)	–	–

**Table 2:**
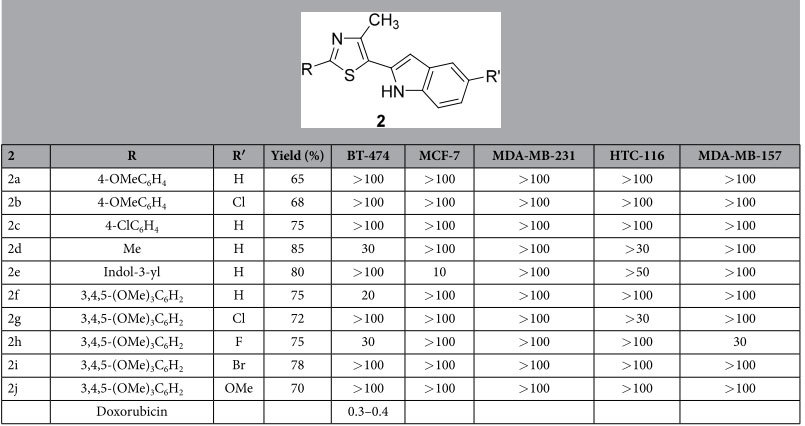
Synthesis and anticancer activity (IC_50_ in μM) of 5-(2^′^-indolyl)thiazoles 2.

**Figure:**
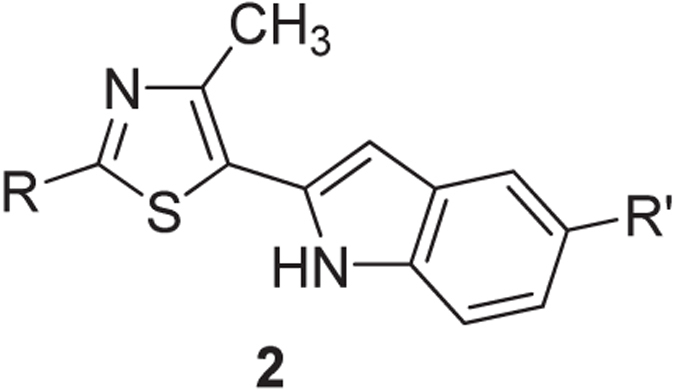


## References

[b1] Zhu J. & Bienaymé H. Multicomponent reactions. 1st Ed. edn, (Weinheim, 2005).

[b2] Polshettiwar V. & Varma R. S. Microwave-Assisted Organic Synthesis and Transformations using Benign Reaction Media. Acc. Chem. Res. 41, 629–639 (2008). 18419142 10.1021/ar700238s

[b3] Gawande M. B., Bonifacio V. D. B., Luque R., Branco P. S. & Varma R. S. Benign by design: catalyst-free in-water, on-water green chemical methodologies in organic synthesis. Chem. Soc. Rev. 42, 5522–5551 (2013). 23529409 10.1039/c3cs60025d

[b4] Gawande M. B., Shelke S. N., Zboril R. & Varma R. S. Microwave-Assisted Chemistry: Synthetic Applications for Rapid Assembly of Nanomaterials and Organics. Acc. Chem. Res. 47, 1338–1348 (2014). 24666323 10.1021/ar400309b

[b5] Browne L. M., Conn K. L., Ayert W. A. & Tewari J. P. The camalexins: new phytoalexins produced in the leaves of camelina sativa (cruciferae). Tetrahedron 47, 3909–3914 (1991).

[b6] Pedras M. S. C., Okanga F. I., Zaharia I. L. & Khan A. Q. Phytoalexins from crucifers: synthesis, biosynthesis, and biotransformation. Phytochemistry 53, 161–176 (2000). 10680168 10.1016/s0031-9422(99)00494-x

[b7] Oka H. . A new topoisomerase-II inhibitor, BE-10988, produced by a streptomycete. J. Antibiot. 44, 486–491 (1991). 1648054 10.7164/antibiotics.44.486

[b8] Ayer W. A., Craw P. A., Ma Y.-t. & Miao S. Synthesis of camalexin and related phytoalexins. Tetrahedron 48, 2919–2924 (1992).

[b9] Dzurilla M., Ružinský M., Kutschy P., Tewari J. & Ková ik V. Application of 2-substituted ethyl isothiocyanates and 2-aminothiols in the synthesis of the analogs of indole phytoalexin camalexin. Collect. Czech. Chem. Commun. 64, 1448–1456 (1999).

[b10] Moody C. & Swann E. Synthesis of the naturally occurring indolequinone BE 10988, an inhibitor of topoisomerase II. J. Chem. Soc., Perkin Trans. 1, 2561–2565 (1993).

[b11] Moody C. J., Swann E., Houlbrook S., Stephens M. A. & Stratford I. J. Synthesis and biological activity of thiazolylindolequinones, analogs of the natural product BE 10988. J. Med. Chem. 38, 1039–1043 (1995). 7699696 10.1021/jm00006a024

[b12] Moody C. J., Roffey J. R., Stephens M. A. & Stratford I. J. Synthesis and cytotoxic activity of indolyl thiazoles. Anti-Cancer Drugs 8, 489–499 (1997). 9215613 10.1097/00001813-199706000-00012

[b13] Jiang B. & Gu X.-H. Syntheses and cytotoxicity evaluation of bis(indolyl)thiazole, bis(indolyl)pyrazinone and bis(indolyl)pyrazine: analogues of cytotoxic marine bis(indole) alkaloid. Bioorg. Med. Chem. 8, 363–371 (2000). 10722159 10.1016/s0968-0896(99)00290-4

[b14] Cheng X. M., Filzen G. F., Geyer A. G., Lee C. & Trivedi B. K. Substituted thiazoles and oxazoles that modulate PPAR activity. WIPO patent WO2003074051 (2003).

[b15] Nowakowski J. 5-Heteroyl indole derivatives. US5409941 (1995).

[b16] Diana P. . Synthesis and antitumor properties of 2,5-bis(3′-indolyl)thiophenes: analogues of marine alkaloid nortopsentin. Bioorg. Med. Chem. Lett. 17, 2342–2346 (2007). 17306531 10.1016/j.bmcl.2007.01.065

[b17] Shaaban M. R., Saleh T. S., Mayhoub A. S., Mansour A. & Farag A. M. Synthesis and analgesic/anti-inflammatory evaluation of fused heterocyclic ring systems incorporating phenylsulfonyl moiety. Bioorg. Med. Chem. 16, 6344–6352 (2008). 18502132 10.1016/j.bmc.2008.05.011

[b18] Schlecker R. & Thieme P. C. The synthesis of antihypertensive 3-(1,3,4-oxadiazol-2-yl)phenoxypropanolahines. Tetrahedron 44, 3289–3294 (1988).

[b19] Liras S., Allen M. P. & Segelstein B. E. A mild method for the preparation of 1, 3, 4-oxadiazoles: Triflic anhydride promoted cyclization of diacylhydrazines. Synth. Commun. 30, 437–443 (2000).

[b20] Masaaki M., Kazuo O., Shinji S., Hiroshi M. & Türk G. H. New thiazolylbenzofuran derivatives, processes for the preparation thereof and pharmaceutical composition comprising the same. EP0528337B1 (1999).

[b21] Kumar D., Sundaree S., Johnson E. O. & Shah K. An efficient synthesis and biological study of novel indolyl-1,3,4-oxadiazoles as potent anticancer agents. Bioorg. Med. Chem. Lett. 19, 4492–4494 (2009). 19559607 10.1016/j.bmcl.2009.03.172

[b22] Kumar D., Kumar N. M., Sundaree S., Johnson E. O. & Shah K. An expeditious synthesis and anticancer activity of novel 4-(3′-indolyl)oxazoles. Eur. J. Med. Chem. 45, 1244–1249 (2010). 20047778 10.1016/j.ejmech.2009.12.024

[b23] Kumar D., Maruthi Kumar N., Chang K.-H. & Shah K. Synthesis and anticancer activity of 5-(3-indolyl)-1,3,4-thiadiazoles. Eur. J. Med. Chem. 45, 4664–4668 (2010). 20692741 10.1016/j.ejmech.2010.07.023

[b24] Kumar D., Narayanam M. K., Chang K.-H. & Shah K. Synthesis of novel indolyl-1,2,4-triazoles as potent and selective anticancer agents. Chem. Biol. Drug Des. 77, 182–188 (2011). 21251232 10.1111/j.1747-0285.2010.01051.x

[b25] Varma R., Kumar D. & Liesen P. Solid state synthesis of 2-aroylbenzo [b] furans, 1, 3-thiazoles and 3-aryl-5, 6-dihydroimidazo [2, 1-b][1, 3] thiazoles from -tosyloxyketones using microwave irradiation. J. Chem. Soc., Perkin Trans. 1, 4093–4096 (1998).

[b26] Jordan A., Hadfield J. A., Lawrence N. J. & McGown A. T. Tubulin as a target for anticancer drugs: agents which interact with the mitotic spindle. Med. Res. Rev. 18, 259–296 (1998). 9664292 10.1002/(sici)1098-1128(199807)18:4<259::aid-med3>3.0.co;2-u

[b27] Hadfield J. A., Ducki S., Hirst N. & McGown A. T. Tubulin and microtubules as targets for anticancer drugs. Prog. Cell Cycle Res. 5, 309–325 (2003). 14593726

